# A public health success: newborn screening for congenital adrenal hyperplasia in a middle-income Brazilian state

**DOI:** 10.1016/j.jped.2025.02.001

**Published:** 2025-03-16

**Authors:** Scott D. Grosse

**Affiliations:** Department of Pediatrics, University of Minnesota, Minneapolis, USA

Newborn screening (NBS) of infants for multiple congenital conditions is widely viewed as one of the most important public health success stories of the past 60 years, although only a minority of infants worldwide currently benefit from public health NBS owing to limited implementation in low- and middle-income countries (LMICs).^1^ It is therefore important to document successful LMIC experiences in the implementation of public health NBS programs, including the lessons learned by programs in addressing resource limitations and logistical challenges.

Most NBS conditions are individually rare inherited disorders. For example, classic congenital adrenal hyperplasia (CAH) due to 21-hydroxylase deficiency (21OHD), is an endocrine disorder occurring between 1 in 10,000 and 1 in 20,000 infants in most populations.^2^ Timely detection and management of the classic salt-wasting (SW) and simple virilizing (SV) forms of 21OHD CAH enable the prevention or mitigation of morbidity and mortality risk in infancy associated with acute adrenal crises, although risks of adverse outcomes remain elevated and require lifelong management.^2^^,^^3^ Even though NBS for CAH has been widely implemented in high-income countries, fewer than half of Latin American countries routinely screen newborns for CAH.^1^

In 2012, Brazil expanded its nationally recommended NBS panel and included CAH. As Brazil has a federal system of government in which state health departments are responsible for the implementation of NBS programs, NBS coverage varies across states.^4^ In this issue of *Jornal de Pediatria*, Barra et al. describe the implementation of public health NBS for CAH in Minas Gerais (MG) state during 2013–2022, the successes and challenges experienced by the state program, and future directions.^5^ The state-run MG NBS program is jointly coordinated by the State Health Department and the NUPAD unit at the Federal University of Minas Gerais (UFMG), which combines operational support for NBS with a program of applied research. The average per capita household income in MG in 2023 was very slightly (1 %) higher than for Brazil overall,^6^ lower than in other Brazilian states from which reports on CAH—NBS have come.^7^ Furthermore, MG has a widely dispersed population and the largest number of municipalities of any state.

The MG NBS program mostly relies on Sistema Único de Saúde (SUS, the Brazilian public health system) outpatient units to collect infant dried blood spot (DBS) specimens at 3–7 days after birth, followed by centralized laboratory testing of DBS specimens^4^, ^5^ and follow-up by the public health clinics. In some Brazilian states, most DBS specimens are collected in hospitals prior to discharge.^7^ The MG program has demonstrated very high coverage of CAH screening using a 17-hydroxyprogesterone (17OHP) immunoassay and subsequent follow-up. The MG program has shown that it is possible to prevent serious short-term outcomes from 21OHD even though screening results are usually reported after 14 days, when infants may have already begun to manifest clinical disease. The authors acknowledge “treatment delay, but with no deaths”.^5^ The absence of recorded deaths during follow-up of varying durations among the 103 infants with SW-CAH in MG is consistent with findings from other Brazilian state NBS programs^7^^,^^8^ and represents an important public health success. The authors report that 22 % of infants with SW-CAH diagnosed through the MG public health program were hospitalized due to an adrenal crisis by the time of the first specialist appointment. The overall percentage of infants with SW-CAH admitted to hospital, including admissions after diagnosis, was not reported. Higher proportions of hospitalizations during infancy have been reported in both unscreened (76 %) and screened (58 %) SW-CAH cohorts in Sao Paulo state, but those estimates cannot be directly compared with the MG findings because of the difference in length of follow-up.^7^ If the hospitalization rate for salt loss among infants with CAH in MG were lower than in other states, it might result from close monitoring of infants with elevated 17OHP levels for signs of salt loss prior to a confirmatory diagnosis; more information would be needed to test that hypothesis.

Barra et al. discuss ways by which the MG program has acted to reduce the number of false-positive (FP) screening results and the resulting burden on families and clinicians, including the use of birthweight (BW)-specific 17OHP screening cutoffs since infants born prematurely, with low birth weights, often have elevated 17OHP values unrelated to CAH. The positive predictive value (PPV) for CAH NBS using 17OHP rose 5-fold from 2.1 % in a pilot screening project during 2007–2008 in which a single cutoff was used,^9^ to 10.5 % during 2013–2021 with BW-specific cutoffs. Similarly, the FP referral rate among all births fell by 80 %, from 0.31 % during the screening pilot to 0.06 % during program implementation.^5^ Other contributing factors to the reduced frequency of referrals for confirmatory testing included using improved assays (removing cross-reacting steroid compounds) and close monitoring of hospitalized premature infants. Experts in high-income countries recommend another approach to achieve a reduced FP screening rate, second-tier testing using liquid chromatography-tandem mass spectrometry (LC-MS/MS).^3^ However, as the second-tier screening approach requires specialized equipment, which is more costly than flow injection MS/MS widely used in NBS, only a minority of CAH screening programs in high-income countries have implemented multi-tier screening using LC-MS/MS.^10^

Although gestational age (GA) of <37 wk, i.e., preterm birth, is superior to BW < 2500 g in predicting elevated risk of 21OHD among newborns, unspecified challenges in obtaining accurate and reliable GA estimates prevented the MG NBS program from using GA to guide referrals until recently.^5^ Many NBS programs worldwide also use low BW as a proxy for preterm birth in part because GA is harder to standardize owing to varying methods of GA estimation. The MG program began using GA estimates to target 17OHP screening cutoffs during the last 6 months of the study, after which the number of referrals for new appointments based on positive screens is said to have fallen by 40 %. It would be of interest to readers to know more about this recent progress. To what extent was the reduction in the number of referrals for diagnostic testing due to the implementation of GA-specific screening cutoffs compared with increased numbers of repeat 17OHP screens performed for hospitalized preterm infants? In addition, documentation of the changes in data systems that allowed for the introduction of GA-specific cutoffs in MG could enable other NBS programs to benefit from lessons learned.

Stratification of estimates of diagnosed CAH cases and FP referrals among preterm births using the WHO GA-based classification—late (34–36 wk), moderate (32–33 wk), very (28–31 wk), and extreme (< 28 wk) preterm birth—might inform a potential refinement of the CAH—NBS referral algorithms for infants born preterm in MG. A recent literature review prepared in conjunction with an analysis of an unscreened CAH case series from Algeria reports that it is rare for infants with confirmed CAH to have GA < 34 wk.^11^ In a published CAH—NBS cohort from MG not included in the review by Ladjouze et al., the lowest GA recorded among infants with confirmed CAH was 36 wk.^9^

The sensitivity of detection of classic 21OHD CAH in the MG screening program may be less than the reported 99.3 %. The sensitivity of screening for classic CAH depends on screening algorithms, the timing of specimen collection, and the length of clinical follow-up of undiagnosed screen-positive infants.^12^ Many infants with 21OHD CAH have 17OHP levels that rise with age;^3^ US NBS programs that routinely request repeat specimens for screening after 1 wk of age report a higher prevalence of classic CAH, especially SV-CAH.^13^^,^^14^ Programs with comprehensive long-term clinical follow-up have documented multiple false negative (FN) screening results, particularly in children with SV-CAH.^15^ Among 106,476 infants screened in MG during the 2007–2008 CAH NBS pilot study, 8 were identified with classic CAH through screening.^9^ After researchers performed *CYP21A2* genotyping at a mean age of 3.4 years for 33 children who continued to have elevated serum 17OHP levels without a clinical diagnosis of CAH, one had a genotype consistent with SV-CAH.^16^ If one were to include the latter child as a subclinical case of classic 21OHD CAH, the sensitivity of NBS for the detection of clinical or subclinical classic CAH in the 2007–2008 pilot study cohort would be 88.9 % (8/9).

Barra et al. suggest that early *CYP21A2* genotyping is “a valuable complement to the 17OHP analysis…although the timeline to complete the molecular protocol might not be suitable for some countries or regions”.^5^ It is important to distinguish between the diagnostic and prognostic utility of *CYP21A2* genotyping and genotyping as a second-tier screening test.^2^^,^^3^ Although *CYP21A2* genotyping of infants and parents has been shown to be clinically useful in the care of infants with confirmed CAH or clinically suspected SW-CAH,^17^, ^18^, ^19^, ^20^ the utility of genotyping as a second-tier NBS test for infants with elevated 17OHP screening results has not been demonstrated,^3^^,^^15^^,^^17^ In addition, it is important to consider the balance of costs and benefits of routine *CYP21A2* genotyping given limited public health resources.

Barra et al. acknowledge opportunities to further improve the practice of CAH NBS in MG by addressing the remaining challenges. One of those challenges is the lack of data on NBS tests and clinical follow-up by the private sector, which accounts for roughly 10 % of all births in the state. To assess the overall performance of NBS in MG would require comprehensive information on outcomes for all infants, regardless of type of health coverage. Another challenge that was acknowledged by the authors is the lack of data in MG on geographic, social, and economic disparities in access to care following screening. Such data would be required to assess the delivery of optimal care at the population level. The MG program reports that increased use of telehealth has facilitated effective access to care throughout the state, and it would be helpful to see further documentation of this successful model of care.

Other challenges in CAH NBS might also be addressed in future work. First, the timeliness of reporting NBS results could be improved. The MG NBS program might consider piloting initiatives aimed at shortening the turnaround time in the reporting of screening results and resulting in earlier diagnoses of CAH and other conditions.^4^ It could also be informative to assess the relative costs of different possible screening and diagnostic strategies for CAH. In particular, a comparison of the added costs of LC-MS/MS second-tier testing with the reduced costs to the public health system and families associated with reductions in the number of referrals for diagnostic evaluation could inform future decisions on the possible implementation of multi-tier testing.

## Conflicts of interest

The authors declare no conflicts of interest.
